# Glioblastoma in the oldest old: Clinical characteristics, therapy, and outcome in patients aged 80 years and older

**DOI:** 10.1093/nop/npad070

**Published:** 2023-10-20

**Authors:** Christina Stadler, Dorothee Gramatzki, Emilie Le Rhun, Andreas F Hottinger, Thomas Hundsberger, Ulrich Roelcke, Heinz Läubli, Silvia Hofer, Katharina Seystahl, Hans-Georg Wirsching, Michael Weller, Patrick Roth

**Affiliations:** Department of Neurology and Brain Tumor Center, University Hospital Zurich and University of Zurich, Zurich, Switzerland; Department of Neurology and Brain Tumor Center, University Hospital Zurich and University of Zurich, Zurich, Switzerland; Department of Neurology and Brain Tumor Center, University Hospital Zurich and University of Zurich, Zurich, Switzerland; Department of Neurosurgery, Clinical Neuroscience Center, University Hospital and University of Zurich, Zurich, Zurich; Inserm, University of Lille, Lille, France; Neuro-Oncology, General and Stereotaxic Neurosurgery Service, University Hospital of Lille, Lille, France; Departments of Oncology & Clinical Neurosciences, Lundin Family Brain Tumor Research Center, Lausanne University Hospital & University of Lausanne, Lausanne, Switzerland; Department of Neurology and Department of Medical Oncology and Haematology, Cantonal Hospital, St. Gallen, Switzerland; Cantonal Hospital Aarau, Aarau, Switzerland; Division of Oncology, University Hospital Basel, Basel, Switzerland; Department of Biomedicine, University of Basel, Basel, Switzerland; Department of Neurology and Brain Tumor Center, University Hospital Zurich and University of Zurich, Zurich, Switzerland; Department of Neurology and Brain Tumor Center, University Hospital Zurich and University of Zurich, Zurich, Switzerland; Department of Neurology and Brain Tumor Center, University Hospital Zurich and University of Zurich, Zurich, Switzerland; Department of Neurology and Brain Tumor Center, University Hospital Zurich and University of Zurich, Zurich, Switzerland; Department of Neurology and Brain Tumor Center, University Hospital Zurich and University of Zurich, Zurich, Switzerland

**Keywords:** chemotherapy, elderly, glioma, radiotherapy, surgery

## Abstract

**Background:**

Incidence rates of glioblastoma in very old patients are rising. The standard of care for this cohort is only partially defined and survival remains poor. The aims of this study were to reveal current practice of tumor-specific therapy and supportive care, and to identify predictors for survival in this cohort.

**Methods:**

Patients aged 80 years or older at the time of glioblastoma diagnosis were retrospectively identified in 6 clinical centers in Switzerland and France. Demographics, clinical parameters, and survival outcomes were annotated from patient charts. Cox proportional hazards modeling was performed to identify parameters associated with survival.

**Results:**

Of 107 patients, 45 were diagnosed by biopsy, 30 underwent subtotal resection, and 25 had gross total resection. In 7 patients, the extent of resection was not specified. Postoperatively, 34 patients did not receive further tumor-specific treatment. Twelve patients received radiotherapy with concomitant temozolomide, but only 2 patients had maintenance temozolomide therapy. Fourteen patients received temozolomide alone, 35 patients received radiotherapy alone, 1 patient received bevacizumab, and 1 took part in a clinical trial. Median progression-free survival (PFS) was 3.3 months and median overall survival (OS) was 4.2 months. Among patients who received any postoperative treatment, median PFS was 3.9 months and median OS was 7.2 months. Karnofsky performance status (KPS) ≥70%, gross total resection, and combination therapy were associated with better outcomes. The median time spent hospitalized was 30 days, accounting for 23% of the median OS. End-of-life care was mostly provided by nursing homes (*n* = 20; 32%) and palliative care wards (*n* = 16; 26%).

**Conclusions:**

In this cohort of very old patients diagnosed with glioblastoma, a large proportion was treated with best supportive care. Treatment beyond surgery and, in particular, combined modality treatment were associated with longer OS and may be considered for selected patients even at higher ages.

Glioblastoma is the most common malignant primary brain tumor in adults and represents almost half of all newly diagnosed malignant brain tumors.^1^ Most patients are diagnosed with glioblastoma in their 60s and the age-adjusted incidence not only peaks among individuals older than 75 years but seems to be rising.^2–4^ Ongoing demographic changes will lead to a growing number of people living up to very high age.^5^ Therefore, very old individuals will account for an increasing proportion of brain tumor patients. As of today, there is no curative therapy for glioblastoma, and survival remains poor.^6^ Higher age is a strong predictor of particularly unfavorable outcome.^7,8^ Older publications report that the median overall survival (OS) declines with age and hardly exceeds 6 months in elderly patients.^9,10^ Among patients over 80 years, the median OS ranges from 2 to 3.5 months.^4,11^ Despite increasing awareness, elderly patients continue to be underrepresented in clinical trials^12–14^ and even such trials that focus on geriatric patients include only a minority of patients beyond the age of 80.^15–19^ The standard treatment for glioblastoma patients up to the age of approximately 70 years consists of maximum safe tumor resection, followed by combined radiochemotherapy with temozolomide.^20^ This treatment is typically well tolerated in younger patients but may impose a higher risk for toxicity in the elderly.^21–23^ This potential vulnerability, but also insecurities regarding treatment efficacy in geriatric patients, may underlie the large proportion of very old patients not undergoing any tumor-specific therapy.^24^ Monotherapy approaches in glioblastoma patients beyond the age of 65 evolved from efforts to develop tolerable treatment strategies for this often frail population and have been implemented in many European countries such as Switzerland and France. Methylation of the O^6^-methylguanine DNA-methyltransferase (*MGMT*) promoter predicts benefit from temozolomide, whereas patients without *MGMT* promoter methylation may be treated with hypofractionated radiotherapy alone.^16,25^ However, there are data suggesting that patients over the age of 80 years with MGMT-unmethylated tumors may have only very limited benefit from radiotherapy alone.^26^ Carefully selected patients, that is, those with a good performance status and methylated *MGMT* promoter, may well qualify for combined radiochemotherapy.^13,19,27^ However, any oncological treatment is associated with additional burden and may result in adverse effects on quality of life because of side effects.^28^ Although the quality of life of elderly glioblastoma patients appears to be mainly determined by tumor progression and not by therapy, treatment-associated decline in quality of life is important to consider in patients with limited survival. Hence, in the absence of clear guidance from prospective trials, optimal management of very old glioblastoma patients remains controversial. The evaluation of unselected patient data may help bridge this gap, reflecting real-life conditions. To shed light on current treatment practice and to identify possible predictors of survival, we therefore analyzed treatment patterns and outcomes of glioblastoma patients aged 80 years or older in a multicentric retrospective cohort study. Considering the relevance of palliative care in this population, we also addressed aspects of supportive treatment and time spent hospitalized.

## Methods

### Study Design

This multicenter retrospective cohort study was conducted in 6 neuro-oncology centers in Switzerland and France (University Hospital Zurich, Cantonal Hospital St. Gallen, CHUV Lausanne University Hospital, Cantonal Hospital Aarau, University Hospital Basel, and Centre Hospitalier Régional Universitaire de Lille). Data collection was approved by the local ethics boards. Automated searches of electronic chart systems identified 107 patients with a follow-up until death, who were histopathologically diagnosed with glioblastoma at the age of 80 years or older between January 2005 and February 2018.

### Variables

All tumors were diagnosed according to the World Health Organization (WHO) criteria at local pathology departments.^29,30^ Tumor progression was determined by local investigator standards and defined as either clinical deterioration or radiological progression on contrast-enhanced magnetic resonance imaging or computed tomography. Progression-free survival (PFS) was defined as the time from surgery to disease progression or death from any cause. Overall survival (OS) was defined as the time from initial surgery until death or last follow-up. Baseline variables included demographics, performance status, and presenting symptoms. Treatment-related variables included the number and extent of surgical interventions (biopsy, subtotal, and gross total resection, ie, complete removal of contrast-enhancing tumor), further treatment and associated adverse events, as well as supportive treatment. Details on the use of psycho-oncological and specialized palliative care services, the time hospitalized, and the place of end-of-life care were also annotated. Tumor-specific data included tumor location, the presence of isocitrate dehydrogenase 1 or 2 (IDH 1/2) mutations (assessed by immunohistochemistry or DNA sequencing), and the *MGMT* promoter methylation status.

### Statistical Analysis

A Karnofsky performance status (KPS) of ≥70% was used as cutoff for a continuing capacity for independent self-care, and a KPS of ≥90% for the presence of minor symptoms only. The log-rank test was applied to compare survival outcomes. A multivariate Cox proportional hazards model was applied to explore factors associated with inferior survival that were univariately significant at the 0.05 level. Statistical tests were 2-tailed and a *P* value of .05 was set as statistically significant. All statistical analyses were performed using Prism 7.04 (GraphPad Software, La Jolla, CA) and IBM SPSS Statistics 27 (IBM Corporation, Armonk, NY) statistical software.

## Results

### Patient Characteristics and Symptoms at Presentation

The median age at diagnosis was 82.4 years (Table 1). Most patients were between 80 and 85 years old (*n* = 89, 83%). There was a slight excess of male patients (*n* = 60; 56%), similar to the sex distribution in younger glioblastoma patients.^1^ Among 90 (84%) patients with available documentation of perioperative KPS, most patients had a KPS higher than 70% (*n* = 60; 67%). The most frequent comorbidities at diagnosis were arterial hypertension (*n* = 51; 51%) and cardiovascular diseases (*n* = 38; 38%), followed by other types of cancer (*n* = 22; 22%), additional neurological disorders (*n* = 15; 15%), and dyslipidemia (*n* = 14; 14%). Diabetes mellitus (*n* = 8; 8%), cerebrovascular (*n* = 9; 9%), and pulmonary diseases (*n* = 8; 8%) were less frequent. Most patients presented with motor deficits (*n* = 51; 53%), followed by psychiatric symptoms and cognitive changes (*n* = 40; 42%) (Table 2). Other frequent findings included aphasia 29 (30%) and seizures (*n* = 25; 26%). Visual disturbances (*n* = 14; 15%), sensory deficits (*n* = 13; 14%), and headaches (*n* = 13; 14%) were less common complaints. In 2 asymptomatic patients, tumors were incidental findings (2%). Other presenting symptoms included nausea, incontinence (*n* = 3 each), weight loss and vertigo (*n* = 2 each), hypacusis, strabism, dysphagia, singultus, and anisocoria (*n* = 1 each).

**Table 1:** Clinical and Histological Characteristics at Diagnosis

	*N* (%)
Age at diagnosis (years): median ± SD	82.4 (±2.5)
80–85	89 (83)
>85	18 (17)
Sex	
Male	60 (56)
Female	47 (44)
Karnofsky performance status^a^	
≤60	30 (33)
70–80	37 (41)
≥90	23 (26)
Comorbidities^b^	
Other malignancy	22 (22)
Cerebrovascular	9 (9)
Other neurological	15 (15)
Cardiovascular	38 (38)
Diabetes	8 (8)
Hypertension	51 (51)
Dyslipidemia	14 (14)
Pulmonary	8 (8)
Other	56 (56)
None	4 (4)
IDH^c^	
Wild type	53 (96)
SEQ	41 (75)
IHC	12 (22)
Mutated	2 (4)
*MGMT* promoter^d^	
Methylated	20 (33)
Unmethylated	41 (67)

Abbreviations: IDH, isocitrate dehydrogenase 1/2; SEQ, IDH status determined by DNA sequencing; IHC, IDH status determined by immunohistochemistry; *MGMT*, O^6^-methylguanine DNA-methyltransferase.

^a^Data available for 90 patients.

^b^Data available for 100 patients.

^c^Data available for 55 patients.

^d^Data available for 61 patients.

**Table 2:** Presenting Symptoms^a^

	*N* (%)
Motor deficit	51 (53)
Psychiatric and cognitive changes	40 (42)
Aphasia	29 (30)
Seizures	25 (26)
Visual disturbance	14 (15)
Headache	13 (14)
Sensory deficit	13 (14)
Other symptoms^b^	15 (16)
Asymptomatic	2 (2)

^a^Data available for 96 patients, some patients presented with more than one symptom or sign.

^b^Including vertigo, falls, hypacusis, incontinence, strabism, dysphagia, singultus, nausea, weight loss, and anisocoria.

### Tumor Characteristics

As expected in this population, almost all tumors that were analyzed harbored no isocitrate dehydrogenase (IDH) mutation (*n* = 53; 96%) (Table 1). In most cases (*n* = 41; 75%), the IDH status was determined by sequencing for IDH 1 and 2. Other samples were only examined by immunohistochemistry (*n* = 12; 22%). Two patients had tumors with an IDH mutation, which would no longer be diagnosed as glioblastoma according to the 2021 WHO classification.^31^ Information regarding the *MGMT* promoter methylation status was available in 61 (57%) patients. An unmethylated *MGMT* promoter was found in 67% of the analyzed samples, whereas the *MGMT* promoter was methylated in 33%.

### Tumor-Specific Treatment

Among the 107 patients included in this study, information on the mode of surgery was available for 100 patients. In 30 patients (30%), subtotal resection was performed, and in 25 patients, gross total resection (25%) was performed, whereas 45 patients (45%) had a biopsy only (Table 3). Data on postoperative first-line treatment were available in 97 patients and included the best supportive care alone in 34 patients (35%). In most cases, this was due to low-performance status (*n* = 19). Furthermore, 10 patients did not wish to receive additional therapy following surgery (Table 3). Radiotherapy with concomitant temozolomide was administered to 12 patients (12%), including 2 patients (2%) who also received maintenance temozolomide (6 and 12 cycles, respectively), radiotherapy alone in 35 patients (36%), and temozolomide alone in 14 patients (14%). Most patients received a radiation dose of 40 Gy, and 5 of these patients had concomitant temozolomide treatment. Five patients (5%) received a dose of 34 Gy, 2 of them with concomitant temozolomide. Six patients (6%) were treated with a dose of 60 Gy. The remaining patients (7%) had differing radiation regimens, all without concomitant chemotherapy. One patient received single-agent bevacizumab as a first-line treatment and 1 patient was enrolled in the ARTE trial and was treated with short-course radiotherapy and bevacizumab.^15^ Fourteen patients (14%) received second-line treatment after tumor progression: 2 patients (2%) underwent re-resection, 1 patient proceeded with bevacizumab, and the other one without any further treatment. The most commonly administered second-line treatment was temozolomide (*n* = 6; 6%). Two patients each received radiotherapy, bevacizumab (which is approved in Switzerland for this indication), or a combination of bevacizumab and an alkylating agent, respectively. Six of the 14 patients (6% of all patients) who received second-line therapy had third-line treatment. Most of these patients received antiangiogenic therapy with bevacizumab (*n* = 4, 4%). Complications during chemotherapy were overall rare (Supplementary Table 1). Thromboembolism was reported in 3 patients (11%) receiving chemotherapy. Clinically meaningful myelotoxicity occurred in 2 patients. One was treated with combined temozolomide-based radiochemotherapy as first-line treatment, and the other one with single-agent temozolomide. Other adverse events included pneumonia (*n* = 1), loss of appetite (*n* = 2), and fatigue (*n* = 1). One patient who received bevacizumab developed arterial hypertension.

**Table 3:** Treatment of the Oldest Glioblastoma Patients

	*N* (%)
Surgery^a^	
Biopsy	45 (45)
Subtotal resection	30 (30)
Gross resection	25 (25)
First-line treatment^b^	
TMZ	14 (14)
RT	35 (36)
RT/ TMZ	10 (10)
RT/TMZ → TMZ	2 (2)
Trial (RT/ Bev)^c^	1 (1)
Bev	1 (1)
None	34 (35)
Patient’s wish	10 (10)
Reduced overall condition	19 (20)
Second-line treatment^b^	
Surgery at recurrence	2 (2)
Surgery alone	1 (1)
Surgery + Alkyl	1 (1)
Bev	2 (2)
Bev + Alkyl	2 (2)
TMZ	6 (6)
RT	2 (2)
Third-line treatment^b^	
Bev	4 (4)
Bev + rindopepimut	1 (1)
Lomustine	1 (1)

Abbreviations: Alkyl, alkylating antineoplastic agent; Bev, bevacizumab; EOR, extent of resection; RT, radiotherapy; RT/TMZ, radiotherapy with concomitant temozolomide; RT/ TMZ → TMZ, radiotherapy with concomitant temozolomide, followed by maintenance treatment with temozolomide; RT/ Bev, radiotherapy with concomitant bevacizumab; TMZ, temozolomide.

^a^Data available for 100 patients.

^b^Data available for 97 patients.

^c^ARTE trial, RT + bevacizumab.

Data regarding the time spent in hospital was available for 92 patients (86%). Among these, the median cumulative time spent hospitalized from diagnosis until death was 30 days (range 4–168 days), which represents approximately a quarter (23%) of their median survival time of 128 days (range 1–1384 days).

### Supportive Treatment and End-of-Life Care

**Figure 1:**
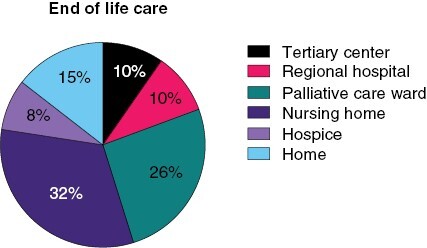
End-of-life care in elderly glioblastoma patients. Places of end-of-life care are indicated.

Supportive therapy was an integral part of the therapeutic management (Supplementary Figure 1). Steroids were applied to almost all patients at some point (*n* = 89; 97% of 92 patients). The vast majority of patients already received steroids before surgery (*n* = 72; 79%). Most patients also had steroids perioperatively (*n* = 79; 86%) and subsequently throughout the clinical course (*n* = 67; 82%). Dexamethasone was the most frequently used drug, followed by methylprednisolone. As more than half of the patients (*n* = 54; 57%) suffered from epileptic seizures, antiepileptic treatment was a crucial part of the supportive treatment regimen. While a quarter of patients presented with seizures at the time of diagnosis (*n* = 25; 27%), 9 patients (10%) developed seizures perioperatively and another third later (*n* = 26; 31%). Although only 57% of patients were reported to have symptomatic epilepsy, 60 patients (65%) were treated with at least 1 antiepileptic drug (AED) suggesting that some patients were treated prophylactically. Levetiracetam was the most frequently used AED (*n* = 41; 67%). Analgesics were used by more than half of the patients (*n* = 50; 57%). Nonsteroidal anti-inflammatory drugs were the most popular pain relievers (n = 45; 90%), followed by highly potent opioids (*n* = 6; 12%). Antiemetics were administered in 30 patients (34%). Mostly 5-HT3 antagonists were used (*n* = 20; 67%), followed by dopamine antagonists (*n* = 9; 30%) and steroids (*n* = 4; 13%). Psycho-oncological support was used by 27 patients (30%). Three patients (3%) needed inpatient admission to a psychiatric ward. Fourteen patients received antidepressant therapy (18%), mostly with a selective serotonin reuptake inhibitor (SSRI). Eleven patients (14%) had an outpatient consultation with a specialized palliative care professional and 26 patients (24%) were admitted to a palliative care ward. End-of-life care was in most cases provided by nursing homes (*n* = 20; 32%) and palliative care wards (*n* = 16; 26%). Nine patients (15%) with known place of death died at home, whereas 6 patients each (10%) died in tertiary center hospitals and peripheral hospitals, respectively. For a large proportion of patients, no information about the place of end-of-life care could be retrieved (*n* = 40; 37%; Figure 1).

### Survival and Associated Factors Among Very Old Glioblastoma Patients

**Figure 2:**
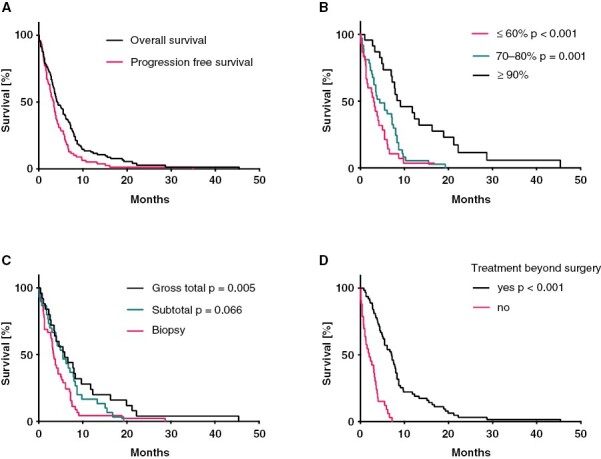
Overall survival (OS) and progression-free survival (PFS) in ≥80-year-old glioblastoma patients. (A) Kaplan–Meier survival curves for OS and PFS. (B) Kaplan–Meier survival curves for OS by Karnofsky performance status. (C) Kaplan–Meier survival curves for OS by extent of tumor resection. (D) Kaplan–Meier survival curves for OS by implementation of postoperative treatment.

**Figure 3:**
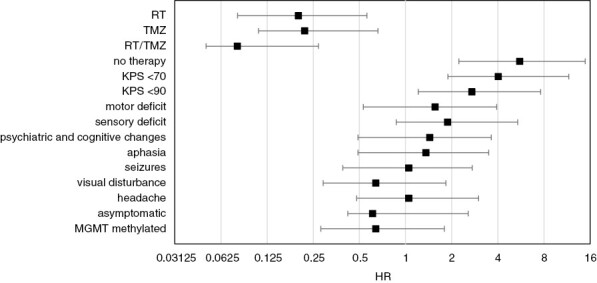
Hazard ratios (HRs) for overall survival (OS) in patient subgroups defined by treatment, clinical characteristics, and *MGMT* promoter methylation status. Univariate Cox regression analyses were performed to identify factors predicting OS. The plots show HRs for death in patient subgroups according to different parameters. *MGMT*, O^6^-methylguanine DNA-methyltransferase; RT, radiotherapy: RT/TMZ, radiotherapy with concomitant temozolomide; TMZ, temozolomide.

The median PFS was 3.3 months, and the median OS was 4.2 months (Figure 2). Median PFS and OS of patients receiving treatment beyond surgery were 3.9 and 7.2 months, respectively. To identify factors that were associated with OS, univariate Cox regression analysis was applied to patient characteristics and treatment factors. KPS was the most powerful predictor of survival (Figures 2 and 3). A KPS of ≤60% was associated with a hazard ratio (HR) of 4.03 (*P* < .001, 95% confidence interval [CI] 2.13–7.61), whereas a KPS of 70%–80%, indicating a condition where autonomous functioning is still possible, resulted in an HR of 2.71 (*P* = .001, CI 1.5– .91), compared to patients with higher KPS (≥90%). Motor deficits were associated with inferior outcome (*P* = .038, HR 1.56, CI 1.03–2.37). A similar finding was observed in patients who presented with sensory deficits (*P* = .047, HR 1.88, CI 1.01–3.50). Other presenting symptoms or specific preconditions, including other types of cancer or cardiovascular disease, were not associated with prognosis. There was also a trend for longer OS of patients with an *MGMT* promoter-methylated tumor; however, this was not statistically significant (*P* = .137, HR 0.64, CI 0.36–1.15). Patients with gross total resection survived longer than patients who underwent a biopsy only (*P* = .005, HR 0.47, 95% CI 0.28–0.80) (Figure 2). However, there was no significant advantage for subtotal resection compared to biopsy only (*P* = .066, HR 0.64, 95% CI 0.39–1.03). Irrespective of the underlying rationale, patients who did not undergo any further treatment after diagnostic surgery, had shorter overall survival (*P* < .001, HR 5.56, 95% CI 3.33–9.29). Comparing different first-line treatment strategies to a palliative approach alone, there was an advantage for a combination of temozolomide and radiation (*P* < .001, HR 0.08, 95% CI 0.03–0.19) compared to temozolomide or radiotherapy alone (TMZ: *P* < .001, HR 0.22, 95% CI 0.11–0.44; RT: *P* < .001, HR 0.20, 95% CI 0.12–0.36).

### Multivariate Analyses

In order to identify relevant factors associated with OS, we applied a multivariate Cox regression model that accounted for established prognostic factors (Table 4). A KPS below 90%, steroid use at presentation, and diagnosis by biopsy versus gross total resection were associated with inferior OS, whereas administration of any postoperative therapy and younger age were associated with longer OS. Taking the *MGMT* promoter methylation and use of temozolomide as first-line treatment into account as well, presenting with a KPS >90% and receiving temozolomide therapy were associated with a favorable outcome (Supplementary Table 2).

**Table 4:** Multivariate Analyses of Inferior Overall Survival

	Hazard Ratio	*P*
Age at diagnosis	0.97 (0.86–1.07)	.489
KPS at presentation		
≥90%	Ref.	
70%–80%	3.64 (1.74–7.60)	.001
≤60%	3.27 (1.46–7.28)	.004
Steroids at presentation: yes vs. no	1.61 (0.86–3.00)	.134
Extent of resection		
Gross total	Ref.	
Subtotal	0.82 (0.38–1.77)	.619
Biopsy	1.82 (1.02–3.25)	.044
Any postoperative therapy: yes vs. no	0.17 (0.09–0.32)	<.001

Abbreviations: KPS, Karnofsky performance status; Ref., reference.

## Discussion

An increasing proportion of glioblastoma patients affects the elderly, a cohort with a particularly poor prognosis, for which there is no broadly accepted standard of care. To shed light on current treatment conventions and possible factors affecting outcomes, we analyzed a multicenter cohort of patients aged 80 years or older. We found that a majority of patients received best supportive care alone. Patients who qualified for tumor-specific treatment had longer overall survival (Figures 2 and 3). Furthermore, a significant fraction of patients spent a considerable part of their remaining lifetime hospitalized, possibly reducing their quality of life. Many patients of our elderly collective presented with psychiatric and cognitive changes. This is in line with previous findings^32^ and stresses the importance of a thorough clinical workup of cognitive impairment and psychiatric symptoms in geriatric patients. Remarkably, most of our very old patients had an initial KPS ≥70%, and KPS was the most powerful predictor of survival. Other baseline features associated with poor outcomes included motor and sensory deficits (Figure 3). Importantly, in our cohort of patients, comorbidities did not influence outcome. Our findings support the use of the KPS as a parameter in the process of treatment allocation. However, in patients over 80 years, a more thorough geriatric assessment, for example, with the G8 screening tool, might be helpful to select candidates for more intense treatment regimens.^33,34^ Furthermore, as this study was limited to patients who underwent a neurosurgical procedure to obtain neuropathological confirmation of the diagnosis, it potentially excluded a subset of elderly patients who were in a too frail state to undergo this first required procedure. However, it remains important to realize that patients in this age category, who were judged fit enough to undergo a surgical procedure, even if it is only a minimally invasive stereotactic biopsy, may present with rapid and significant deterioration of their general health condition, highlighting the importance of the evaluation of the performance status in elderly patients.

One possible explanation for the exceptionally poor prognosis of elderly glioblastoma patients might be that tumors in this age group represent a distinct glioblastoma subgroup but no age-specific molecular signature has been identified.^35^ Because of their distinct biology, IDH-mutant tumors, which are rare in elderly patients, are now no longer called glioblastoma.^31^ In our cohort, 96% of the patients with a known IDH status did not display a mutation in the IDH genes. The finding that 2 patients in our cohort had IDH-mutant tumors may warrant IDH testing, including DNA sequencing, also in old patients, once specific treatment options for these patients become available. The *MGMT* promoter methylation status was distributed as expected in our very old collective with one-third of the patients having methylated tumors. There was a trend for increased OS in these patients.

In line with previous data,^36^ our study revealed that very old patients are less likely to have extensive surgery, with 45% undergoing biopsy only. The reservation toward more aggressive resection might seem reasonable, given the increased risk of complications in the elderly.^37^ In line with previous reports,^38^ our data support the implementation of gross total resection, whenever considered safe and possible. Although KPS may help to identify eligible patients, further studies on the role of tumor resection in old glioblastoma patients are needed, with a particular focus on quality of life.^39^

For younger glioblastoma patients, radiotherapy with concomitant and maintenance treatment with temozolomide has become the standard of care.^12^ In our cohort of old patients, therapy beyond surgery consisted of radiotherapy only in most patients. Considering that two-thirds of these had a KPS ≥70%, and 6% an *MGMT* promoter-methylated tumor, the number of patients receiving combined radiochemotherapy followed by maintenance temozolomide was low. Of note, approximately a third of our patients were treated before prospective studies focusing on elderly glioblastoma patients were available.^15–19^ At least some patients, particularly with *MGMT-*methylated tumors, may have benefitted from temozolomide as mono- or combination therapy.^26,40^ Moreover, among patients who received chemotherapy with temozolomide only, 50% had a KPS ≥70% and might have qualified for combination therapy.^19^ Interestingly, 21% of these patients had an unmethylated *MGMT* promoter, pointing to a limited benefit of alkylating therapy in these cases.^26^ Our data demonstrate that temozolomide chemotherapy is feasible even in patients older than 80 years. One-third of all patients did not undergo any tumor-specific treatment. Postsurgical treatment, however, was associated with longer OS. Although this difference might again be due to selection bias, it is in line with previous data on patients older than 70 years^23^ and even 80 years, respectively.^26,38^ However, on multivariate analysis accounting for KPS, gross total resection and combined radiochemotherapy were still associated with longer OS. Considering our findings with current studies at hand, patient outcomes might have been better with more aggressive treatment approaches. The exploration of the best therapeutic approach requires randomized data in this population of patients. Carefully selected patients with a good performance status may well qualify for combined radiochemotherapy even at an age above 80 years or single-modality therapy according to the *MGMT* status. Finally, more research is needed to better understand patient wishes and expectations regarding the available treatments as well as therapy-associated side effects.

Quality of life of glioma patients is often impaired by hospitalization.^41^ Our study reveals that elderly glioblastoma patients spend a significant amount of their remaining lifetime as inpatients. In this regard, the association of surgery and tumor-specific treatment with shorter hospitalization deserves particular attention in elderly patients.^28^

Up to 70% of glioblastoma patients will suffer from seizures at some point during the course of their disease.^42,43^ There was a trend for poor survival among patients with perioperative seizures in our collective. In general, elderly patients should preferably be treated with newer AED at the lowest effective dose and primary prophylactic treatment should be avoided.^44,45^ In line with these recommendations, levetiracetam was by far the most frequently used AED in this cohort. Although only 57% of our patients were diagnosed with epilepsy, 65% were treated with at least 1 drug, suggesting that there might be an unwarranted overuse of AED as a primary prophylaxis. Symptomatic treatment with glucocorticoids was also broadly applied in our patient cohort. Elderly patients are particularly prone to side effects and the use of steroids has been identified as an independent negative prognostic factor in glioblastoma patients.^46–48^ Therefore, particularly in this collective, steroid doses and treatment duration should be kept to a minimum.

Anxiety and depression are common neuropsychiatric symptoms in elderly glioblastoma patients.^49^ In our cohort, a significant proportion of patients were taking antidepressants, mostly SSRI. More studies are needed to explore if this is actually the best treatment option for elderly patients. Only a third of our patients had psycho-oncological counseling, suggesting that clinicians should be sensitized for the need of psycho-oncological support. Moreover, the benefit of integrating a specialist palliative care team should not be withheld from elderly patients.^50^ Finally, patients’ satisfaction at the end of life is also associated with dying at the preferred place. In our cohort, end-of-life care was mostly provided by nursing homes and palliative care wards. Only 15% of the patients died at home and 20% died at hospitals. It needs to be investigated to what extent this corresponded to patients’ wishes, but this contrasts experiences from other countries, where most glioblastoma patients die at home, which may partially be due to differences in healthcare systems.^51^ Possible explanations might be the extraordinarily fast disease progression in elderly glioblastoma patients, as well as their reduced capacity to express their will, hampering the organization of adequate end-of-life care. For these patients, it might be important that neuro-oncologists help facilitate the dignified end-of-life care by implementing advanced care planning and consulting caregivers.^52^

In summary, our analysis of this rather neglected population of very old glioblastoma patients indicates that more efforts are needed to improve the management of these patients in order to improve their prognosis as well as quality of life.

## Supplementary material

Supplementary material is available online at *Neuro-Oncology* (https://academic.oup.com/neuro-oncology).

npad070_suppl_Supplementary_Figures_1

npad070_suppl_Supplementary_Tables_1-2
