# Supplementary material for: Glioblastoma in the oldest old: Clinical characteristics, therapy, and outcome in patients aged 80 years and older
Source: Neurooncol Pract. 2023 Oct 20;11(2):132–41. doi: 10.1093/nop/npad070 (PMC10940826; doi:10.1093/nop/npad070)
Supplement: npad070_suppl_Supplementary_Figures_1 [file npad070_suppl_supplementary_figures_1.pptx]

## Slide 1
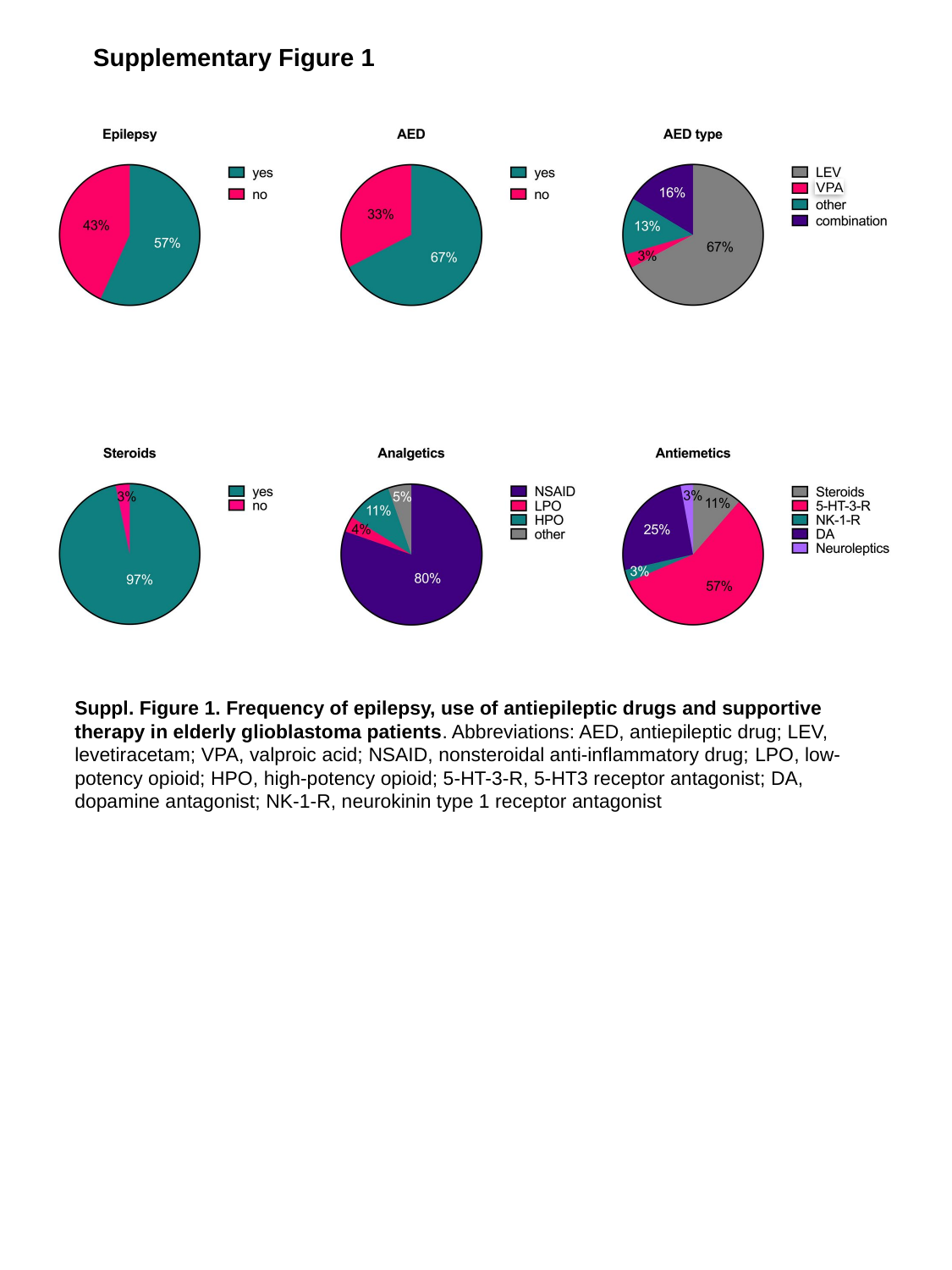

Supplementary Figure 1
VPA
Suppl. Figure 1. Frequency of epilepsy, use of antiepileptic drugs and supportive therapy in elderly glioblastoma patients. Abbreviations: AED, antiepileptic drug; LEV, levetiracetam; VPA, valproic acid; NSAID, nonsteroidal anti-inflammatory drug; LPO, low-potency opioid; HPO, high-potency opioid; 5-HT-3-R, 5-HT3 receptor antagonist; DA, dopamine antagonist; NK-1-R, neurokinin type 1 receptor antagonist
