# Supplementary material for: Glioblastoma in the oldest old: Clinical characteristics, therapy, and outcome in patients aged 80 years and older
Source: Neurooncol Pract. 2023 Oct 20;11(2):132–41. doi: 10.1093/nop/npad070 (PMC10940826; doi:10.1093/nop/npad070)
Supplement: npad070_suppl_Supplementary_Tables_1-2 [file npad070_suppl_supplementary_tables_1-2.docx]

**Supplementary Tables**

**Suppl. Table 1**

**Adverse events during systemic therapy**

| **Treatment** | **Myelotoxicity** | **Bleeding** | **Thromboembolism** | **Other AE** |
| --- | --- | --- | --- | --- |
| TMZ | 1 | 1 | 2 | Fatigue  Loss of appetite (N=2) |
| RT / TMZ | 1 |  | 1 | Pneumonia |
| Bev |  |  |  | Hypertension |

**Suppl. Table 1. Abbreviations:** AE: adverse event; TMZ: temozolomide; RT / TMZ: radiotherapy with concomitant temozolomide; Bev: Bevacizumab

**Suppl. Table 2**

**Multivariate analyses of inferior overall survival including temozolomide treatment and *MGMT* status**

|  | **Hazard ratio** | **p** |
| --- | --- | --- |
| **Age at diagnosis** | 1.01 (0.87 – 1.17) | 0.913 |
| **KPS at initial presentation**  ≥ 90%  70 - 80%  ≤ 60% | Ref.  6.79 (2.40 – 19.18)  3.60 (1.45 – 8.94) | < 0.001  0.006 |
| **Steroids:** yes vs. no | 0.89 (0.836- 2.21) | 0.798 |
| **Extent of resection**  Gross total  Subtotal  Biopsy | Ref.  1.64 (0.79 – 3.43)  0.47 (0.16 – 1.44) | 0.186  0.187 |
| **Temozolomide as first line therapy:** yes vs. no | 2.93 (1.05 – 8.20) | 0.040 |
| ***MGMT* promoter methylation:** yes vs. no | 1.14 (0.43 – 2.10) | 0.792 |

**Suppl. Table 2. Abbreviations:** TMZ: temozolomide; KPS: Karnofsky performance status; MGMT: O^6^-methylguanine DNA-methyltransferase
